# Prediction of intravesical recurrence of non-muscle-invasive bladder cancer by evaluation of intratumoral Foxp3^+^ T cells in the primary transurethral resection of bladder tumor specimens

**DOI:** 10.1371/journal.pone.0204745

**Published:** 2018-09-27

**Authors:** Ryosuke Murai, Yasushi Itoh, Susumu Kageyama, Misako Nakayama, Hirohito Ishigaki, Kazuo Teramoto, Mitsuhiro Narita, Tetsuya Yoshida, Keiji Tomita, Ken-ichi Kobayashi, Akinori Wada, Masayuki Nagasawa, Shigehisa Kubota, Kazumasa Ogasawara, Akihiro Kawauchi

**Affiliations:** 1 Department of Pathology, Shiga University of Medical Science, Otsu, Shiga, Japan; 2 Department of Urology, Shiga University of Medical Science, Otsu, Shiga, Japan; University of South Alabama Mitchell Cancer Institute, UNITED STATES

## Abstract

Patients with a history of non-muscle-invasive bladder cancer sometimes have recurrence of tumors after transurethral resection of bladder tumor treatment. To find factors related to the recurrence of non-muscle-invasive bladder cancer, we examined tissue specimens taken at transurethral resection of bladder tumor as an initial treatment. We revealed the association between prognosis of non-muscle-invasive bladder cancer and infiltration of Foxp3^+^ T cells that suppress anti-tumor immunity in 115 primary non-muscle-invasive bladder cancer patients retrospectively identified and followed for at least 3 months after primary transurethral resection. In immunohistological staining, we counted the number of cells positive for CD3 and positive for CD3 and Foxp3 together and calculated the percentage of Foxp3^+^ T cells among the CD3^+^ T cells. The recurrence-free survival rate was calculated by the Kaplan-Meier method, and a Cox regression analysis of recurrence factors was performed. The median (interquartile range) percentage of Foxp3^+^ T cells in all cases was 17.1% (11.9, 11.4–23.3%). Compared by risk stratification, it was 11.4% (10.4, 7.8–18.2%) in the low-risk group (n = 32), 16.8% (12.6, 11.6–24.2%) in the intermediate-risk group (n = 45), and 22.0% (9.7, 16.4–26.1%) in the high-risk group (n = 38). The Kaplan-Meier survival analysis indicated that the Foxp3^+^ T cell high group (≥ 17.1%) had a worse RFS rate than did the low group (< 17.1%) (P = 0.006). In multivariate analysis, the percentage of Foxp3^+^ T cells was an independent risk factor for intravesical recurrence (hazard ratio 2.25). Thus, peritumoral Foxp3^+^ T cell infiltration was correlated to risk stratification and recurrence-free survival. Therefore, the percentage of Foxp3^+^ T cells in tumor specimens may predict a risk for intravesical recurrence.

## Introduction

Bladder cancer is the eleventh most common cancer and the seventh most common in men who are newly diagnosed, according to a worldwide review [1]. Non-muscle-invasive bladder cancer (NMIBC) comprises 75% of primary bladder cancer cases and has a mortality rate that is lower than that of muscle-invasive bladder cancer. However, the 5-year-recurrence rates and 5-year-progression rates after treatment for NMIBC are in the ranges of 50% to 70% and 10% to 30%, respectively [2]. Since the high recurrence rate in NMIBC impairs the quality of life in many patients, reducing the recurrence rate is clinically important. Therefore, we need to find a new biomarker to classify patients who may have a high recurrence risk.

Recent advances in cancer immunology research indicate that the cancer microenvironment, such as invasion of immunosuppressive cells and cytotoxic immune cells, affects the development of cancer [3]. Regulatory T (Treg) cells are a subpopulation of T cells with highly immunosuppressive function, which are characterized by expression of forkhead box P3 (Foxp3) in the nuclei [4]. In muscle-invasive bladder cancer, some evidence supports a correlation between invasion of Foxp3^+^ T cells into cancer tissue and patient prognosis [5,6], but a relationship between Foxp3^+^ T cells and the recurrence of NMIBC, which is an earlier stage of bladder cancer, has not been evaluated previously. In addition, in the previous studies, Treg cells were identified in immunohistochemical staining for Foxp3 alone. This method might overestimate the number of Treg cells since the other type of the cells express Foxp3 [7–10].

In the present study, we examined the relationship between infiltration of Foxp3^+^ T cells into peritumor tissues and NMIBC recurrence using immunostaining for Foxp3 together with CD3 (a part of T-cell antigen receptor) to identify Treg cells more precisely than did the previous studies [7–10]. We found that patients with high percentages of Foxp3^+^ T cells in peritumor tissues had higher recurrence rates than did those with low percentages of Foxp3^+^ T cells after primary transurethral resection of bladder tumor (TURBT). This finding suggests that the percentage of Foxp3^+^ T cells in TURBT specimens may be used for prognostic prediction.

## Material and methods

### Patients and tissue samples

We retrospectively collected samples from 115 primary bladder cancer patients who had received TURBT and who were followed-up for at least 3 months after the operation at the Shiga University of Medical Science from January 1, 2001, to June 30, 2009. The longest follow-up period was 120 months. These patients comprised 92 males (80%) and 23 females (20%) with a median age of 68.0 years (range: 27–88 years). The histological diagnosis was non-muscle-invasive urothelial carcinoma in all patients. The main clinicopathological parameters of patients are shown in Table 1. Follow-up data were collected from all patients. The median follow-up period was 26.0 months (range 3–120 months). The recurrence-free survival (RFS) time was defined as the interval between primary TURBT and a time point when recurrence was found with cystoscopy. Risk stratification was evaluated according to the 2016 American Urological Association / Society of Urologic Oncology guidelines [11]. Briefly, the low-risk group was low grade (LG) solitary Ta ≤ 3 cm. The intermediate-risk group was solitary LG Ta > 3 cm or LG multifocal Ta or high grade (HG) Ta ≤ 3 cm or LG T1. The high-risk group was HG T1 or HG Ta > 3 cm (or multifocal) or any carcinoma *in situ* or any variant histological types. The clinical outcomes of the three groups are shown in Table 2. As normal controls, we collected 14 samples of bladder tissue from autopsy cases without any cancer. This study was approved by the Local Research Ethics Committee of Shiga University of Medical Science (No. 27–117).

**Table 1:** Patient characteristics.

Category	Number of patients	Number of recurrent patients
Total	115	58
Age, median (range, years)	68.0 (27–88)	
Months of follow-up, median (range)	26.0 (3–120)	
Gender (%):		
Male	92 (80.0)	47
Female	23 (20.0)	11
Smoking (%):		
Yes	30 (26.1)	12
No	17 (14.8)	12
Unknown	68 (59.1)	34
pT stage (%):		
pTa	70 (60.9)	38
pT1	39 (33.9)	20
pTis	6 (5.2)	0
Pathological WHO grade (%):		
low grade	78 (67.8)	44
high grade	37 (32.1)	14
Risk stratification (%):		
low-risk	32 (27.8)	17
intermediate-risk	45 (39.1)	27
high-risk	38 (33.0)	14
Adjuvant intravesical treatment (%):		
None	60 (52.2)	37
THP	32 (27.8)	17
BCG	23 (20.0)	4
Percentage of Foxp3^+^ T cell (%):		
< 17.1	58 (50.4)	24
≥ 17.1	57 (49.6)	34

**Table 2:** Three risk groups and clinical outcomes.

Risk stratification	Number of patients	Adjuvant intravesical treatment	Number of patients	Number of recurrent patients (%)
low-risk	32	none	31	16 (51.6)
		THP	1	1 (100)
intermediate- risk	45	none	23	15 (65.2)
		THP	22	12 (54.5)
high-risk	38	none	6	6 (100)
		THP	9	4 (44.4)
		BCG	23	4 (17.4)

### Immunohistochemistry and immunofluorescence

For histological analysis, 3-μm-thick formalin-fixed paraffin-embedded tissue sections were stained with hematoxylin and eosin. For immunohistochemical and immunofluorescence staining, formalin-fixed paraffin-embedded tissues were deparaffinized by a standard method, followed by heat-based antigen retrieval in citrate solution (pH 9.0) and pretreated with 0.3% hydrogen peroxide. Non-specific binding of antibody was blocked with Block Ace (DS Pharma Biomedical, Suita, Japan). Next, tissue sections were incubated with anti-Foxp3 primary antibody (Abcam ab20034, 1:1000) overnight. After washing of slide glasses, horseradish peroxidase conjugated anti-mouse Ig secondary antibody (Simple stain MAX-PO(M), Nichirei Bioscience) was added and incubated for 60 min. After washing, Tyramide-FITC reagent (TSA plus kit working solution OPAL520 (FITC), PerkinElmer Life and Analytical Sciences, Boston, MA) was incubated for 10 min according to the manufacturer’s instructions. After washing, tissues underwent the antigen retrieval step for 15 min by microwave in pH 6 sodium citrate solution, and then, tissue sections were incubated with anti-CD3 primary antibody (Dako M7254, 1:500) overnight followed by staining with the secondary antibody for 60 min and incubation with Tyramide-Cy3 reagent (TSA plus kit working solution OPAL570 (Cy3), PerkinElmer) for 10 min. Then, for counter-staining, 4’, 6-diamidino-2-phenylindole was incubated for 5 min. For a comparative purpose, tissue sections stained with the mouse IgG_1_ isotype control (Dako X0931) for the primary antibodies were prepared.

### Measurement of the percentage of Foxp3^+^ T cells

For systematic counting, 5 high-power fields (HPFs) in TURBT specimens and 2–5 high-power fields in biopsy specimens were randomly selected in tumor stroma areas under a confocal microscope (FV1000 OLYMPUS microscope, Tokyo, Japan.) at magnification with 40 × objective lens. We counted the number of cells positive for CD3 (mostly CD3^+^ T cells) and positive for both CD3 and Foxp3 (Foxp3^+^ T cells) and calculated the percentage of Foxp3^+^ T cells in the CD3^+^ T cells.

### Statistical analysis

Statistical analyses were performed using the statistical package SPSS 22.0 (SPSS, Inc., Chicago, IL, USA) and the EZR software (Saitama Medical Center, Jichi Medical University, Saitama, Japan), which is a graphical interface for R (The R Foundation for Statistical Computing, Vienna, Austria). Continuous data were expressed as median (interquartile range), and we used the Mann-Whitney test to compare the continuous variables. The Cox’s proportional hazards model was used to perform univariate and multivariate regression analyses of potential prognostic recurrence factors. Baseline variables with P < 0.20 in univariate analysis were included in the multivariable models. The Kaplan-Meier method and the log-rank test were employed to determine the RFS. The cut-off value at receiver operating characteristics curve (ROC) analysis was calculated as the Youden index.

## Results

### Infiltration of Foxp3^+^ T cells in NMIBC tissues at TURBT

**Fig 1:**
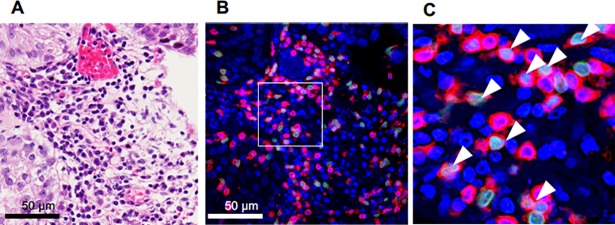
TURBT specimens taken from patients with NMIBC. Representative figures are shown. (A) Hematoxylin and eosin staining. (B) Fluorescent immunohistochemical staining, red: CD3, green: Foxp3, blue: DAPI. (C) A magnified image of the white box in (B). Arrowheads: Foxp3^+^CD3^+^ cells.

**Fig 2:**
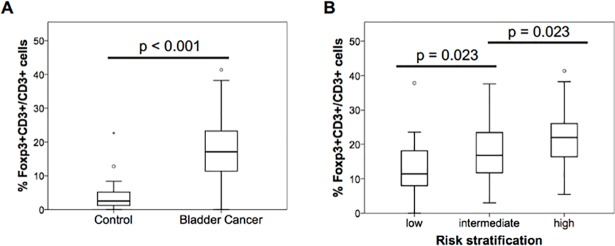
Percentage of Foxp3^+^CD3^+^ T cells in TURBT specimens. (A) Comparison of median (interquartile range) of the percentage of Foxp3^+^ cells among CD3^+^ cells in 14 non-bladder cancer controls and 115 TURBT specimens of NMIBC (P < 0.001, Mann-Whitney test). (B) Comparison of median (interquartile range) of the percentage of Foxp3^+^ cells in CD3^+^ cells in three risk groups (P < 0.001, Kruskal-Wallis test).

We examined the percentage of Foxp3^+^ and CD3^+^ T cells in 115 NMIBC specimens collected at TURBT. Cells positive for both Foxp3 and CD3 (Foxp3^+^ T cells) were detected mainly in the tumor stroma (Fig 1). In the specimens, the median and interquartile range of the percentage of Foxp3^+^ cells among CD3^+^ cells were 17.1% (11.9, 11.4–23.3%), while those in the control group without cancer were 2.6% (4.9, 1.1–6.0%) (P < 0.001). (Fig 2A).

### The relationship between the clinicopathological features and the percentage of Foxp3^+^ T cells

We analyzed the percentage of Foxp3^+^ T cells together with a pathological WHO grade and T stage (Table 3). The percentage of Foxp3^+^ T cells in tumors was positively related to clinicopathological stages diagnosed after TURBT. Also, among the three groups at low-risk, intermediate-risk and high-risk divided by risk stratification of NMIBC, the median percentage of Foxp3^+^ T cells progressively increased according to increased risk (Fig 2B).

**Table 3:** Relationship between clinicopathological features and the percentage of Foxp3^+^CD3^+^ cells.

Clinicopathological features	Median percentage of Foxp3^+^CD3^+^ cells (interquartile range)	p value
Gender:		
Male	18.0 (12.8, 12.0–25.0)	0.02
Female	12.7 (12.1, 7.9–20.0)	
Age (year):		
< 68	18.0 (11.7, 11.5–23.2)	0.502
≥ 68	16.4 (12.4, 11.1–23.5)	
Smoking		
Yes	18.0 (14.1, 11.3–24.7)	0.939
No	16.9 (12.2, 10.7–22.4)	
T Stage:		
Ta	13.3 (12.9, 8.9–21.8)	< 0.001
T1	21.3 (9.7, 16.4–26.1)	
Grade:		
low grade	14.1 (13.1, 9.3–22.4)	< 0.001
high grade	21.3 (9.1, 16.7–25.8)	
Risk stratification:		
low-risk	11.4 (10.4, 7.8–18.2)	< 0.001
intermediate-risk	16.8 (12.7, 11.6–24.3)	
high-risk	22.0 (9.7, 16.4–26.1)	

### ROC analysis of predictors of NMIBC recurrence

ROC analysis was performed on age, the number of CD3^+^ cells, the number of Foxp3^+^ cells, and the percentage of the Foxp3^+^ T cells. The area under the curves (AUCs) of age, the number of CD3^+^ cells, the number of Foxp3^+^ cells, and the percentage of the Foxp3^+^ T cells were 0.525, 0.552, 0.578, and 0.603, respectively. Since the percentage of Foxp3^+^ T cells showed the highest value of them, the best threshold for prediction of recurrence was 17.98 of the percentage of Foxp3^+^ T cells, which was close to the median percentage of FoxP3^+^ T cells in CD3^+^ cells in the tissues obtained at TURBT (17.1%) (Fig 2A). Therefore, this value was used for dividing patients into two groups with the percentage of Foxp3^+^ T cells in the following analysis. In addition, the AUCs of the percentage of the Foxp3^+^ T cells on the basis of the grade classification were 0.630 and 0.691 for the low and high grades, respectively. The AUCs of the percentage of the Foxp3^+^ T cells on the basis of the risk stratification of NMIBC were 0.522, 0.691, and 0.734 for the low-risk, the intermediate-risk, and the high-risk groups, respectively. Thus, the percentage of Foxp3^+^ T cells in the TURBT specimens predicts the recurrence, especially in the higher risk groups.

### The relationship between the percentage of Foxp3^+^ T cells and the patients’ prognoses

In order to investigate prognostic factors to predict the recurrence, univariate and multivariate Cox regression analyses were performed (Table 4). In the univariate analysis, the RFS rates were significantly correlated with addition of intravesical instillation therapy and negatively correlated with the percentage of Foxp3^+^ T cells. The Cox regression multivariate analysis showed that a high percentage of Foxp3^+^ T cells and the absence of adjuvant intravesical therapy and non-smoker were risk factors for intravesical recurrence, although smoking history of more than half of the patients in the present study were unknown.

**Table 4:** Univariate and multivariate Cox regression analysis predicting recurrence-free survival rates after primary TURBT.

	Univariate		Multivariate	
	HR^a^ (95%CI^b^)	P-value	HR (95%CI)	P-value
Gender:				
Male	1.0 (Reference)			
Female	0.87 (0.45–1.68)	0.681	-^c^	-
Age (year):				
< 68	1.0 (Reference)			
≥ 68	0.77 (0.46–1.30)	0.329	-	-
Smoking				
Yes	1.0 (Reference)		1.0	
No	2.0 (0.90–4.45)	0.091	2.74 (1.16–6.48)	0.021
T Stage:				
Ta	1.0 (Reference)			
T1	0.91 (0.53–1.57)	0.739	-	-
Grade:				
Low	1.0 (Reference)		1.0 (Reference)	
High	0.62 (0.34–1.13)	0.119	0.86 (0.26–2.83)	0.807
Risk stratification:				
Low	1.0 (Reference)		-	-
Intermediate	1.03 (0.56–1.90)	0.922	-	-
High	0.59 (0.29–1.20)	0.148	-	-
Adjuvant intravesical therapy:				
No	1.0 (Reference)		1.0 (Reference)	
Yes	0.44 (0.25–0.75)	0.003	0.97 (0.27–0.35)	0.000
Percentage of Foxp3^+^ T cells:				
< 17.98	1.0 (Reference)		1.0 (Reference)	
≥ 17.98	2.03 (1.21–3.43)	0.008	4.45 (1.71–11.5)	0.002

^a^ hazard ratio

^b^ 95% confidence interval

^c^ not calculated

**Fig 3:**
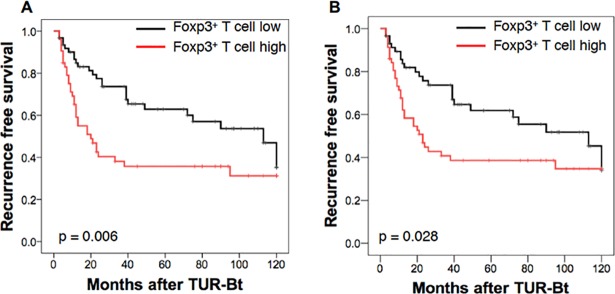
Recurrence-free survival (RFS) rates after TURBT for NMIBC in patients with low and high percentages of Foxp3^+^ cells among CD3^+^ cells. (A) Foxp3^+^ T cell low (< 17.98%) and high (≥ 17.98%) populations are divided on the basis of ROC analysis. Black line: patients with low Foxp3^+^ cells, n = 62. Red line: patients with high Foxp3^+^ cells, n = 53. The difference is significant (P = 0.006) in the log-rank test. (B) Foxp3^+^ T cell low and high populations are divided on the basis of the median (17.1%). Black line: patients with low Foxp3^+^ cells, n = 58. Red line: patients with high Foxp3^+^ cells, n = 57. The difference is significant (P = 0.028) in the log-rank test.

In order to investigate a relationship between the percentage of Foxp3^+^ T cells and prognosis of NMIBC, a Kaplan-Meier curve analysis by the log-rank test was performed (Fig 3A). The patients with a high percentage of Foxp3^+^ T cells in the tumor (≥ 17.98%) had a lower RFS rate than did those with a low percentage of Foxp3^+^ T cells (< 17.98%). The median recurrence-free periods were 20 months and 113 months for the patients with high and low percentages of Foxp3^+^ T cells, respectively (P = 0.006). The similar results were obtained in the Kaplan-Meier curve analysis on the basis of the median percentage of Foxp3^+^ cells in the TURBT specimens (Fig 3B). Thus, the percentage of Foxp3^+^ T cells in the TURBT tissues are corelated with the recurrence free survival rates and recurrence-free periods.

### Subgroup analysis according to risk stratification and intravesical treatment

**Fig 4:**
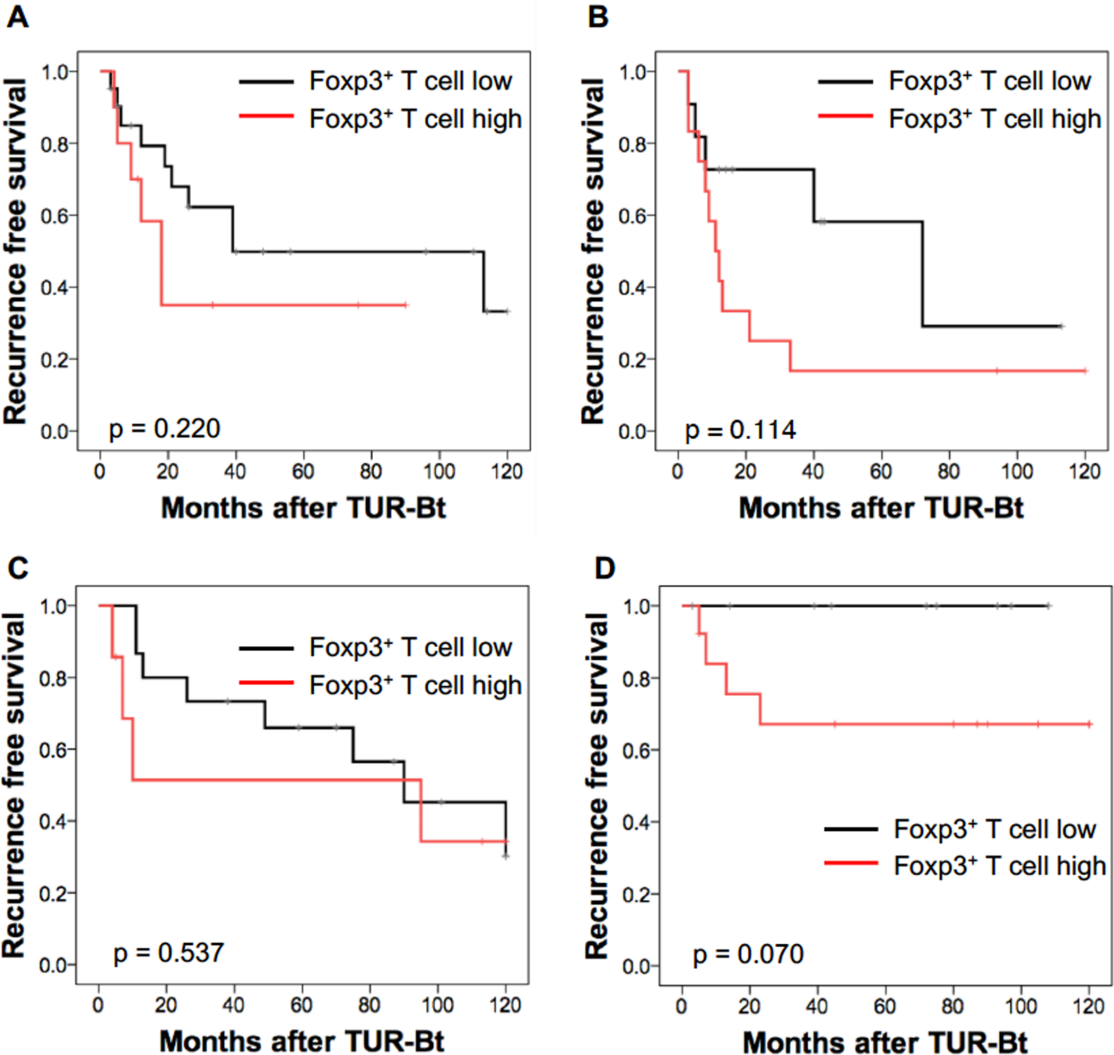
Recurrence-free survival (RFS) rates after TURBT for NMIBC in patients with low (< 17.98%) and high (≥ 17.98%) percentages of Foxp3^+^ cells among CD3^+^ cells by sub-groups according to risk stratification and adjuvant intravesical treatment. (A) RFS rates after TURBT for low-risk NMIBC patients without adjuvant intravesical treatment. Black line: patients with low Foxp3^+^ cells (n = 21). Red line: patients with high Foxp3^+^ cells (n = 10). P = 0.220, log-rank test. (B) RFS rates after TURBT for intermediate-risk NMIBC patients without adjuvant intravesical treatment. Black line: patients with low Foxp3^+^ cells (n = 11). Red line: patients with high Foxp3^+^ cells (n = 12). P = 0.114, log-rank test. (C) RFS rates after TURBT for intermediate-risk NMIBC patients with intravesical THP treatment. Black line: patients with low Foxp3^+^ cells (n = 15). Red line: patients with high Foxp3^+^ cells (n = 17). P = 0.537, log-rank test. (D) RFS rates after TURBT for high-risk NMIBC patients with intravesical BCG treatment. Black line: patients with low Foxp3^+^ cells (n = 10). Red line: patients with high Foxp3^+^ cells (n = 13). P = 0.070, log-rank test.

The Kaplan-Meier curve analysis by the log-rank test was performed on subgroups according to risk stratification and selected intravesical treatment (Fig 4). Thirty-one patients in the low-risk group and 23 patients in the intermediate-risk group received only follow-up observation: the patients with a high percentage of Foxp3^+^ T cells had a lower RFS rate than did those with a low percentage of Foxp3^+^ T cells, but the difference was not statistically significant (Fig 4A and 4B). In the intermediate-risk group, in which 22 out of 45 patients received intravesical instillation of pirarubicin (THP), there was no difference in the RFS rates between patients with a high percentage of Foxp3^+^ T cells and those with a low percentage of Foxp3^+^ T cells (Fig 4C). In the high-risk group, in which 23 out of 38 patients received intravesical instillation of Bacillis Calmette-Guérin (BCG), there was no recurrence in the patients with a low percentage of Foxp3^+^ T cells, whereas the recurrence was detected in the patients with a high percentage of Foxp3^+^ T cells, although the difference was not statistically significant probably due to the low number of patients (Fig 4D). Thus, the percentage of Foxp3^+^ T cells in the tumor tissue seems to be related to sensitivity to intravesical BCG in the high-risk NMIBC.

### Comparison of the CD3^+^ Foxp3^+^ T cells between before and after intravesical BCG treatment

**Fig 5:**
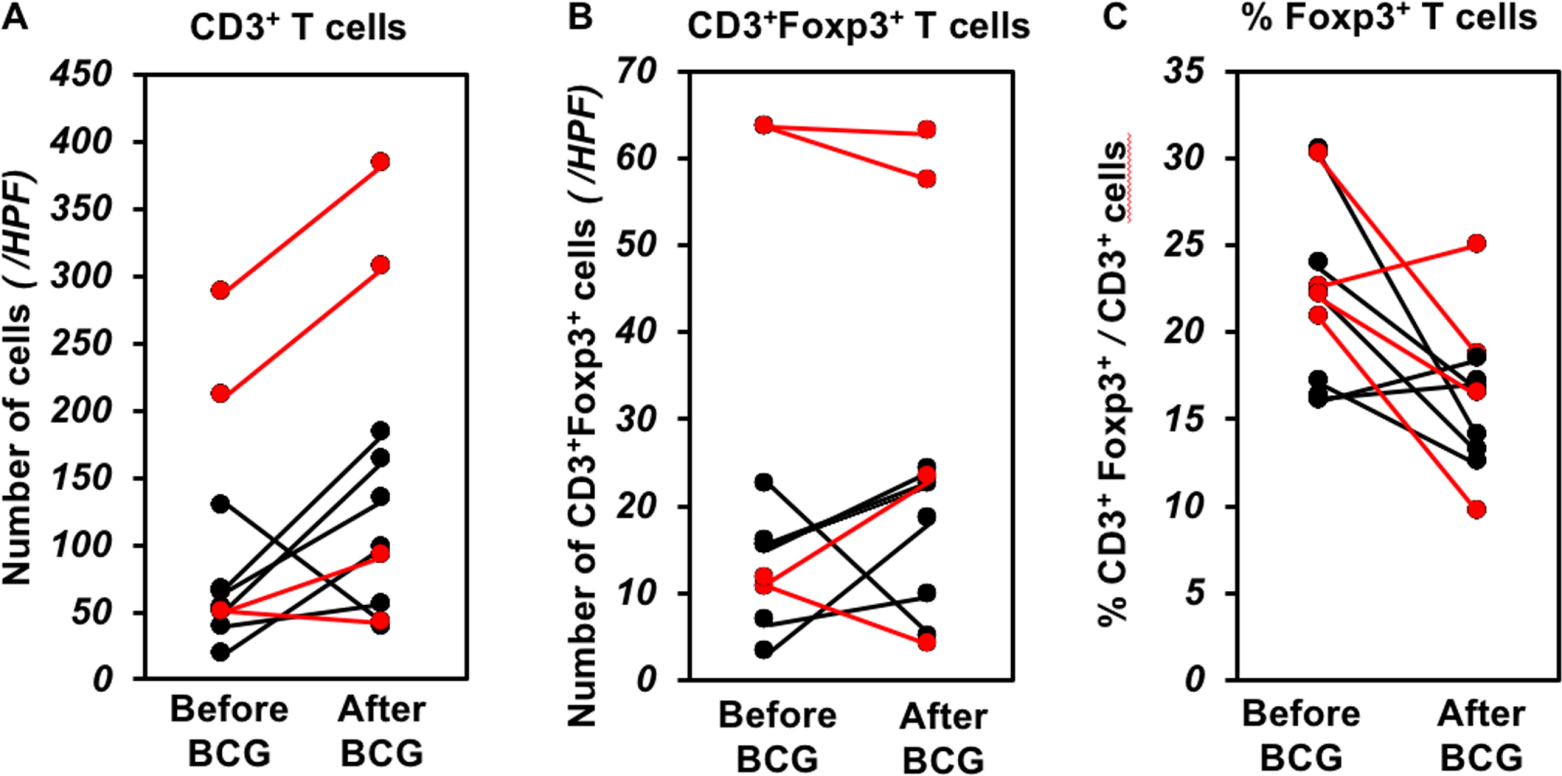
Comparison of CD3^+^ T cells and Foxp3^+^CD3^+^ T cells between before and after intravesical BCG treatment. The average cell numbers in a high-power field (HPF) of biopsy samples after BCG are calculated on the basis of the cell numbers in 2–5 HPFs of stroma areas in each specimen. The cell numbers before BCG are calculated in the TURBT specimens. (A) the number of CD3^+^ cells/HPF, (B) the number of Foxp3^+^CD3^+^ cells/HPF, (C) the percentage of Foxp3^+^CD3^+^ cells in CD3^+^ cells. Red: patients with recurrence (n = 4), black: patients without recurrence (n = 6). (A) The median (interquartile range) of the number of CD3^+^ T cells are 130.7 (179.5, 50.7–230.2) before BCG treatment and 200 (247.6, 794–327) after BCG treatment in 4 patients with recurrence (P = 0.25, Wilcoxon signed rank sum test). The median (interquartile range) numbers of CD3^+^ T cells are 58.4 (24.8, 42.7–67.5)/HPF before BCG treatment and 116.5 (89.2, 67.1–156.2)/HPF after BCG treatment in patients without recurrence (P = 0.219, Wilcoxon signed rank sum test). (b) The median (interquartile range) numbers of Foxp3^+^CD3^+^ T cells are 37.4 (52.3, 11.2–63.5) /HPF before BCG treatment and 40.3 (40.5, 18.3–58.8) /HPFs after BCG treatment in 4 patients with recurrence (P = 0.875, Wilcoxon signed rank sum test). The median (interquartile range) numbers of Foxp3^+^ CD3^+^ T cells are 15.3 (7.1, 8.6–15.7) /HPF before BCG treatment and 20.3 (10.9, 11.8–22.7) /HPF after BCG treatment in patients without recurrence (P = 0.438, Wilcoxon signed rank sum test). (c) The median (interquartile range) percentages of Foxp3^+^CD3^+^ T cells are 22.3% (2.8, 21.7–24.5%) before BCG treatment and 17.5% (5.6, 14.7–20.3%) after BCG treatment in 4 patients with recurrence (P = 0.25, Wilcoxon signed rank sum test). The median (interquartile range) percentages of Foxp3^+^CD3^+^ T cells are 19.7% (7.0, 16.4–23.4%) before BCG treatment and 15.3% (3.6, 13.3–16.9%) after BCG treatment in patients without recurrence (P = 0.156, Wilcoxon signed rank sum test).

Since intravesical BCG treatment had an effect on prevention of recurrence in the high-risk group in which the average percentage of Treg cells in the tumor tissue was higher than those of the low and intermediate groups, we examined the number of Treg cells after intravesical BCG treatment in 10 biopsy samples including 4 recurrent tumors and 6 normal mucosae without recurrence. After BCG treatment, the number of CD3^+^ T cells was increased in the tissues with and without recurrence, whereas the number of CD3^+^Foxp3^+^ T cells was not changed (Fig 5A and 5B). As a result, the percentage of Foxp3^+^ T cells was decreased after intravesical BCG treatment (Fig 5C).

## Discussion

In the present study, we performed a quantitative analysis on infiltration of Treg cells in the NMIBC tissue and revealed a relationship between the percentage of Treg cells in the tumor tissue and the recurrence rate after TURBT of NMIBC: the high frequency of recurrent bladder cancer was observed in patients whose TURBT specimens contained a high percentage of Foxp3^+^CD3^+^ cells. This observation suggests that Foxp3^+^CD3^+^ cells around the tumor contribute to tumor progression. Furthermore, our results indicate that NMIBC induces accumulation of Treg cells around the tumor tissue because the percentage of Foxp3^+^ cells among the CD3^+^ cells in TURBT specimens was higher than that in normal bladder tissue.

In order to identify Treg cells in NMIBC specimens, we stained tissues for CD3 together with Foxp3. In the previous studies using immunohistochemical staining, Treg cells were identified only by expression of the transcription factor Foxp3 [12–15]. However, since it has been reported that Foxp3 protein was expressed in tumor cells and lymphocytes other than Treg cells [7–10], authentic Treg cells might not be detected by staining only for Foxp3. Therefore, we performed co-staining for CD3 and Foxp3 in order to identify Treg cells more precisely in the present study than in previous reports.

Treg cells have been known to suppress anti-tumor responses [16]. It has been reported that infiltration of Treg cells in the tumor tissue was related to a poor prognosis of advanced cancers, including renal cell carcinoma [12], breast cancer [13] and diffuse large B-cell lymphoma [14]. However, in colorectal cancer, the opposite result has been reported [17]. In the urothelial carcinoma, although the relationship between the infiltration of Treg cells and prognosis of invasive bladder cancer has been controversial, recent reports indicate a poor prognosis in patients with severe infiltration of Treg cells in tumor tissues [5,6,15]. Since there are few reports published on Treg cells and prognosis of patients with NMIBC, we examined whether infiltration of Treg cells in the NMIBC tissue is associated with recurrence of bladder cancer. Our results indicate that among all clinical factors the intravesical recurrence rate was most strongly related to the percentage of Treg cells in the tumor tissue (Table 4). This suggests that Treg cells infiltrated in NMIBC tissue have an adverse effect on patients’ prognoses. Therefore, the percentage of Treg cells in tumor tissues taken at TURBT would be a prediction factor for intravesical recurrence.

Although the high-risk NMIBC group contained severe Treg-infiltration in the tumor tissue, this group had a lower recurrence rate than did low-risk and intermediate-risk groups. Since most patients in the high-risk NMIBC group were treated with the intravesical BCG adjuvant, it was considered that the adjuvant intravesical BCG treatment had a great effect on reduction of the recurrence rate in the high-risk group, which was selected on the basis of the risk-stratification of NMIBC [11]. This is concordant with the relationship between the intravesical recurrence rate and the adjuvant intravesical treatment in Table 4. Therefore, it is speculated that intravesical BCG treatment impairs the immune-suppressive function of Treg cells, especially in the low Treg population of the high-risk group, as reported that a high count of Treg cells was a risk factor for NMIBC recurrence after BCG treatment [10].

On the other hand, it was reported that CD4^+^ Treg cells and CD8^+^ Treg cells were increased in tuberculosis patients and after BCG vaccination [18]. Furthermore, Treg cells suppressed activation of BCG-specific CD4^+^ T cells [19]. However, our results indicate that non-Treg CD3^+^ cells, presumably effector T cells, but not Treg cells, were increased in the bladder tissues after BCG treatment and the percentage of Treg cells were decrease as a result, although the difference was not statistically significant probability due to the low number of the patients. To solve this difference in effects of BCG on Treg cells in the bladder tissues from tuberculosis and BCG vaccination, we plan to study the molecular mechanisms and factors involved in attracting Treg cells to the tumor tissue. In the future, this information may help to make it possible to influence quantitatively Treg infiltration among patients.

## Conclusions

The percentage of Foxp3^+^ T cells in specimens of NMIBC taken at TURBT was significantly associated with the recurrence free survival rate and was suggested as a useful prognostic indicator.
